# Brain-derived neurotrophic factor and neuroimaging in pediatric patients with sickle cell disease

**DOI:** 10.1038/s41390-023-02513-5

**Published:** 2023-02-11

**Authors:** Asmaa A. Mahmoud, Sameh A. Abd El Naby, Ahmed S. Abdelgawad, Marwa Sabry Rizq, Nahla M. S. Abd El Hady

**Affiliations:** 1 Department of Pediatrics—Faculty of Medicine, Menoufia University, Shebin Elkom, Egypt; 2 Department of Clinical Pathology, National Liver Institute, Menoufia University, Shebin Elkom, Egypt; 3 Department of Pediatrics, National Liver Institute, Menoufia University, Shebin Elkom, Egypt

## Abstract

**Background:**

The risk of neurological complications is increased in children with sickle cell disease (SCD), such as silent cerebral infarction (SCI) and stroke. Brain-Derived Neurotrophic Factor (BDNF) is a nerve growth factor associated with elevated transcranial Doppler (TCD) velocities and increased risk of stroke in SCD patients. So, we assessed the BDNF level in children with SCD and its relation to neurological complication as silent stroke.

**Methods:**

A comparative cross-sectional study was conducted on 40 patients with SCD, recruited from the Hematology Unit, Pediatric Department, Menoufia University Hospital, and 40 healthy children as controls. Laboratory investigations including BDNF were done. TCD was done for all patients and Magnetic Resonance Imaging (MRI) was done on high-risk patients.

**Results:**

BDNF levels were significantly higher in children with SCD than in controls with a significant relation to TCD findings. There was a statistically significant diagnostic ability of BDNF in the prediction of SCD complications as its sensitivity was 89.5%, specificity (95% CI) was 80% with a cut-off point >0.69, AUC = 0.702, and *p* = 0.004).

**Conclusion:**

Serum BDNF levels were higher in sickle disease patients who had abnormal transcranial Doppler. BDNF had a significant diagnostic ability in the detection of SCD complications.

**Impact:**

- Silent stroke is a very serious complication in children with sickle cell disease, so regular follow up should be every six months.
- BDNF is considered a potential biomarker for stroke risk prediction in patients unable to receive TCD.

## Introduction

SCD is caused by a single base pair mutation (Glu6Val) in the β globin gene. The most common genotype is homozygous hemoglobin SS (HbSS), and common heterozygous condition is Sβ0-thalassemia.^1^

Neurological complications such as silent cerebral infarctions (SCI), overt hemorrhagic and ischemic stroke are increased in the pediatric SCD population.^2^ Repeated vaso-occlusion in sickle cell anemia (SCA) results in acute pain and end-organ ischemic injury such as stroke.^3^ In SCA, ischemic stroke incidence is four times higher in children 2–15 years old^4^ Children with SCA may also develop SCI, without overt stroke.^5^

Neurotrophins play a pivotal role in the development, functioning, survival, and plasticity of the nervous system. BDNF is a widely distributed neurotrophin in the mammalian CNS; its purification has an important role in mammalian brain development, physiology, and pathology.^6^

BDNF is thought to participate by inhibiting apoptosis and stimulating sprouting and neuronal reorganization. The cellular actions of BDNF were mediated through tyrosine kinase receptor B (TrkB) and by p75NTR (p75 neurotrophin receptor), a member of the tumor necrosis factor receptor superfamily.^7^ BDNF has been associated with neuroprotection in ischemic brain injury so, it has prognostic and diagnostic prediction of brain injury.^8,9^

## Methods

### Study design

This comparative cross-sectional study included 40 patients with SCD (they were 22 patients with HbSS and 18 patients with Sβ0-thalassemia diagnosed by hemoglobin electrophoresis) recruited from the hematology unit, pediatric department, Faculty of Medicine, Menoufia University, and 40 healthy volunteers as a control group during the period from April 2021 to April 2022. Patients with other hemoglobinoapathies and other chronic hemolytic anemia were excluded. Informed written consent was obtained from the guardian of all participants. The Institutional Review Board (IRB) of the Menoufia Faculty of Medicine approved the study (ID: 10/2020 PEDI 12). All participants were subjected to complete history taking and physical and clinical examination and laboratory investigations including complete blood count, serum ferritin and BDNF. All SCD patients were on hydroxyurea therapy with good compliance.

### Sample collection

Blood samples of 10 ml were withdrawn from participants during their admission to the hospital by vaso-occlusive crisis under complete aseptic conditions and then were divided into appropriate vacutainer tubes as follows: Two EDTA tubes each contained 2 mL of whole blood for CBC and hemoglobin electrophoresis. Two plain vacutainer tubes each contained 3 mL of blood that was used for serum separation into aliquots to be assayed later for biochemical investigations and BDNF serum level assay.

Serum ferritin was measured according to the electro-chemiluminescence immunoassay “ECLIA” procedure by Cobas 6000 (e 601 modules). CBC was done using a cell counter, Sysmex XT1800i Automated Hematology Analyzer. BDNF serum levels were assayed using a double-antibody sandwich ELISA Kit (by SunRed biotechnology company, cat. No: 201-12-1303, China). The assay of BDNF was performed according to the instructions manual of the kit manufacturer and BDNF concentrations were detected spectrophotometrically using an ELISA microtiter plate reader (ELX800, BioTek Instruments, Inc, SN: 1502175).

### Neuroimaging study

#### Transcranial doppler ultrasound

All patients investigated with color Doppler ultrasound named ESAOTE my lab 60 with 75 and m5s transducer, the transducer created the gray scale image at a frequency between 5.0–12 MHz and Doppler at frequency 5.0 MHz the depth of the image set at 10 cm/s, sample volume set at 1 mm to 2 mm and the color sensitivity and frame rate are optimized.

Velocities were used to classify subjects into one of three exclusive categories: According to Time Averaged Mean Flow (TAMmx) (Normal: TAMmx less than 170 or equal cm H2O- Conditional: TAMmx 170–199 cm H2O- High risk: TAMmx more than 200 cm H2O.^10^

#### Magnetic Resonance Imaging (MRI)

High-risk TCD patients underwent MRI for detection of any radiological evidence of a stroke at the Radiology Department, Faculty of Medicine, Menoufia University using MR TOSHIBA Vantage 1.5 Tesla using NV head coil, and all patients were found normal.

### Sample size calculation

According to Lance et al.^11^ at power 80% and CI 95%, sample size was calculated and found to be 40 subjects in each group with case to control ratio 1:1.

### Statistical analysis

Data were analyzed using IBM SPSS statistics version 20 (SPSS Inc., Chicago, IL). Chi-square test was used to examine relationship between qualitative variables. For quantitative data, Student t-test was used to compare two groups. Kruskal–Wallis test was used to analyze more than two groups as appropriate. The receiver operator characteristic (ROC) curve graphically represents relationship between sensitivity and specificity at different cut-off points for BDNF. Multiple linear regression was carried out to investigate relationship between the dependent variable BDNF and independent variables. A *p*-value < 0.05 was considered significant.

## Results

Socio-demographic data of the studied groups were reported in (Table 1). There was a statistical significant difference between both groups regarding the consanguinity (*p* < 0.001). Regarding the laboratory findings, there was a significant decrease in both hemoglobin level and hematocrit. There was a significant increase in white blood cell count, platelet count, serum ferritin, and BDNF as in (Table 2).

**Table 1:** Socio-demographic data of studied groups.

Variables	Case No = 40		Control No = 40	Test of sig.	*P* value
Age (Years)					
Mean ± SD	9.86 ± 4.74		9.43 ± 4.16	*t*	*p* = 0.689
Range	2–18		2–18	0.402	
Median	9		9		
Sex				*χ*^2^	
Male	22 (55%)		21 (52.5%)		*p* = 1.0
Female	18 (45%)		19 (47.5%)	0.0	
Consanguinity				*χ*^2^	
Yes	21 (52.5%)		5 (12.5%)		*p* < 0.001
No	19 (47.5%)		35 (87.5%)	15.664	
Weight (Kg)				*t*	
Mean ± SD	29.04 ± 13.38		30 ± 12		*p* = 0.8
Range	11–58		14–60	0.51	
Median	25		37.5		
Height (cm)				*t*	
Mean ± SD	128 ± 24		145 ± 22		*p* = 0.965
Range	85–176		100–175	−0.4	
Median	122		123		
BMI (Kg/m^2^)				*t*	
Mean ± SD	17.09 ± 2.61		17.55 ± 2.46		*P* = 0.7
Range	12.25–24		13–26	0.5	
Median	16.8		18		
SCD patients	No.	%			
Sickle cell anemia	22	55	----------------	----------	---------
Sickle thalassemia	18	45			

*SD* standard deviation, *t* student’s t test, *χ*^*2*^ Chi-square test, *BMI* Body mass index.

**Table 2:** Comparison of the laboratory data in the two studied groups.

Variables	Case No = 40	Control No = 40	Test of sig.	*P* value
Hb (gm/dl)				
Mean ± SD	9.2 ± 1.4	11.3 ± 1.2	*t* = −6.668	*p* < 0.001*
Median (Min–Max)	9.1 (6.3–11.2)	11.2 (11.5–13.0)		
Hct (%)				
Mean ± SD	27.3 ± 4.5	32.0 ± 4.5	*t* = −4.387	*p* < 0.001*
Median (Min–Max)	26.6 (18.5–34.7)	33.0 (22.9–37.6)		
MCV (fl)				
Mean ± SD	75.3 ± 7.9	73.2 ± 5.3	*t* = 1.307	*p* = 0.196
Median (Min–Max)	76.1 (73.4–80.3)	73.5 (70–79.9)		
MCH (Pg)				
Mean ± SD	26.5 ± 4.1	23.4 ± 2.1	*t* = 0.17	*P* = 0.92
Median (Min–Max)	26.0 (21.0–30.5)	23.2 (22.7–29.2)		
Platelets (×10^3^/mm^3^)				
Mean ± SD	352.5 ± 202.0	230.3 ± 35.8	*t* = 3.523	*p* = 0.001*
Median (Min–Max)	288 (163–758)	229 (172–286)		
WBCs (×10^3^/mm^3^)				
Mean ± SD	10.2 ± 4.9	7.8 ± 1.5	*t* = 2.734	*p* = 0.008*
Median (Min–Max)	8.6 (4.8–24.5)	7.6 (5.8–10.3)		
Serum ferritin (ng/ml)				
Mean ± SD	933.24 ± 941	44.5 ± 22	*t* = 3.38	*p* < 0.001*
Median (Min–Max)	754 (18.1–3860)	50 (35.5–57)		
Serum BDNF (ng/ml)				
Mean ± SD	12.83 ± 2.95	1.15 ± 0.85	*t* = 2.353	*p* = 0.002*
Median (Min–Max)	2.19–15	0.62–2.36		

*Hb* hemoglobin, *Hct* hematocrit, *MCV* mean corpuscular volume, *MCH* mean corpuscular hemoglobin, *WBCs* white blood cells, *BDNF* brain-derived neurotrophic factor.

An asterisk symbol “*” denotes significant *p* values.

Transcranial Doppler (TCD) findings were normal in 9 cases, conditional in 26 cases, and high-risk in 5 cases. MRI was done for the five cases that had high risk and showed normal findings. Conditional and high risk TCD patients received blood transfusion for 3–6 months. A statically significant difference was found between patients with normal TCD versus conditional and high-risk findings regarding BDNF as shown in (Table 3).

**Table 3:** Comparison between transcranial Doppler ultrasound among studied patients and its relation to BDNF.

Variables	Cases = 40	
	No	%
Transcranial Doppler		
Normal (≤170 cm/s)	9	22.5
Conditional (170–199 cm/s)	26	65
High risk (>200 cm/s)	5	12.5

Variables	Serum BDNF (ng/ml)			*P* value
	Mean ± SD	Median (Min–Max)	Test of Significance	
Transcranial Doppler				
Normal (≤ 170 cm/s)	1.36 ± 0.81	1.1 (2.19–2.36)	H = 12.439	p1 = 0.037*
Conditional (170–199 cm/s)	2.47 ± 1.01	2.7 (2.84–13.77)		p2 = 0.01*
High risk (>200 cm/s)	3.24 ± 0.98	3.28 (4.06–15)		p3 = 1.0

H; Kruskal Wallis test, Pairwise comparison was done using Wilcoxon Ranked test.

p1; *p* value between normal and conditional.

p2; *p* value between normal and high risk.

p3; *p* value between conditional and high risk.

An asterisk symbol “*” denotes significant *p* values.

**Fig. 1:**
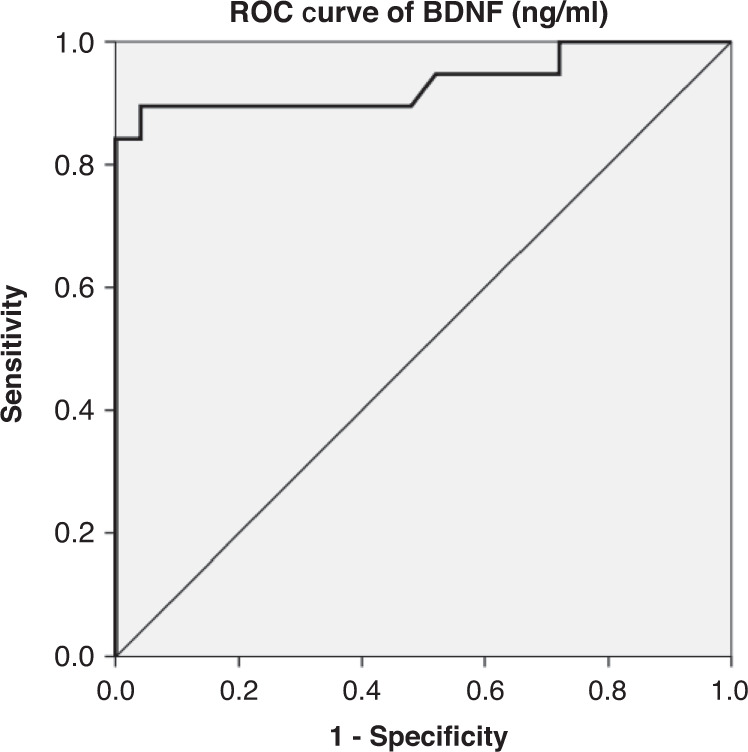
ROC curve analysis of serum BDNF among studied patients.

ROC curve analysis of BDNF for the diagnostic and prognostic ability of BDNF to detect SCD complications with AUC = 0.702, a cut-off point of >0.69, 89.5% sensitivity, and 80% specificity (*p* = 0.004) as in (Table 4 and Fig. 1). Multivariate analysis revealed that abnormal TCD findings were significant for contributing to the prediction of BDNF which increased by 1.45 in patients who have abnormal TCD findings compared to patients with normal TCD findings, 95% CI (0.743 to 2.16) as in (Table 5).

**Table 4:** The ROC curve analysis of serum BDNF for prediction of stroke in SCD patients.

AUC	*P* value (95% CI)	Cut-off point	Sensitivity	Specificity	PPV	NPV	Accuracy
0.702	0.004 0.577–0.822	>0.69	89.5%	80%	77.6%	90.8%	83.9

*AUC* Area under the curve, *PPV* positive predictive value, *NPV* negative predictive value.

**Table 5:** Multiple linear regression analysis to predict the effect of the independent contribution of different factors on BDNF.

Predictors	Standardized Coefficients Beta	*P* value	95% Confidence Interval
Age (year)	0.041	0.83	(−0.08 to 0.1)
Sex^A^	−0.050	0.75	(−0.8 to 0.58)
HbS	0.186	0.334	(−0.157 to 0.448)
Transcranial Doppler^B^	0.638	0.0001*	(0.743 to 2.16)

Dependent Variable: BDNF.

A ref; female.

B ref; normal Doppler.

An asterisk symbol “*” denotes significant *p* values.

## Discussion

Based on hematological investigations, the current study showed a highly significant difference as regards Hb, Hct, platelets, and WBCs. Serum ferritin and BDNF were increased in patients than in controls. Elevated BDNF levels were found in SCD children with both conditional and high-risk TCD compared to controls.

Previous study included 70 children with SCD and 40 children as a control group reported normocytic normochromic anemia with leukocytosis and thrombocytosis in the patient group. Serum ferritin was higher in patients than in the control group.^12^

Lance et al.^13^ reported that BDNF levels were significantly higher in children with SCD in comparison to healthy children. Chambliss et al.^14^ illustrated that BDNF levels were elevated 3 fold in SCD children compared to healthy controls.

Certain studies illustrated the association between elevated serum BDNF and ischemic and traumatic brain injury.^8,15^

Hyacinth et al.^16^ illustrated higher BDNF levels in SATCD (SCA with abnormal TCD compared with SNTCD (SCA with normal TCD) and controls. Also, serum BDNF level was higher in SNTCD (SCA with normal TCD) than in controls.

Higher serum levels of BDNF have been associated with high TCD velocity in children with SCD. Also, BDNF was positively correlated with the volume of the infarction, so it is involved in the pathophysiology of cerebrovascular disease in SCD.^17^

Elevated BDNF level in children with SCA is an adaptive/protective response to reduce cell death during periods of cerebral ischemia caused by abnormal cerebral blood flow (CBF).^18,19^

Based on TCD findings in this study, 9 SCD patients had normal findings, 26 of them had conditional findings and 5 had high-risk TCD.

Sabarense et al.^20^ found 45 SCD patients with high-risk TCD. Only two of them had a stroke. In France, 92 SCD children had abnormal TCD and no patient had a stroke.^21^ In the US, 88 SCD children study, 46 children had normal TCD, 17 children had conditional TCD and 19 children had high-risk TCD and none of them had a stroke.^22^ A Brazilian study reported 15 patients with abnormal TCD under chronic transfusion for primary prevention of stroke.^23^

ROC curve analysis of BDNF for the diagnostic and prognostic ability of BDNF to detect SCD complications with AUC = 0.702, a cut-off point of 0.802, 89.5% sensitivity, and 80% specificity. Multiple linear regression analysis revealed that abnormal TCD is significant for contributing to the prediction of BDNF which increased by 1.45 in patients who have abnormal TCD compared to patients with normal TCD, 95% CI (0.743 to 2.16).

The ROC curve analysis of BDNF was sensitive (AUC = 0.8) and considered a good predictor of abnormal CBF velocity as indicated by high TCD velocity measurement. Logistic regression modeling showed that elevated BDNF level was significantly associated with increased odds of having abnormally high CBF velocity.^16^

The limitation of our study was small sample size, but this is explained by decreased number of patients who had sickle cell disease in our country.

## Conclusion

BDNF levels increased in SCD patients with conditional and high-risk TCD. So, BDNF may be a potential biomarker for stroke risk prediction in patients unable to receive TCD.

## Data Availability

The datasets generated during and/or analyzed during the current study are available from the corresponding author on reasonable request.
